# Executive Functioning in Alcoholics Following an mHealth Cognitive Stimulation Program: Randomized Controlled Trial

**DOI:** 10.2196/jmir.2923

**Published:** 2014-04-17

**Authors:** Pedro Gamito, Jorge Oliveira, Paulo Lopes, Rodrigo Brito, Diogo Morais, Diana Silva, Ana Silva, Sara Rebelo, Marta Bastos, Alberto Deus

**Affiliations:** ^1^Lusophone University of Humanities and TechnologiesLisbonPortugal; ^2^COPELABS-Cognition and People-Centric Computing LabsLisbonPortugal; ^3^São João de Deus InstituteSintraPortugal

**Keywords:** alcohol, addiction, cognitive stimulation, executive function, mobile health, serious games

## Abstract

**Background:**

The consequences of alcohol dependence are severe and may range from physical disease to neuropsychological deficits in several cognitive domains. Alcohol abuse has also been related to brain dysfunction specifically in the prefrontal cortex. Conventional neuropsychological interventions (paper-and-pencil cognitive stimulation training) have a positive effect but are time-consuming, costly, and not motivating for patients.

**Objective:**

Our goal was to test the cognitive effects of a novel approach to neuropsychological intervention, using mobile technology and serious games, on patients with alcohol dependence.

**Methods:**

The trial design consisted of a two-arm study assessing the cognitive outcomes of neuropsychological intervention with mobile serious games (mHealth) versus control (treatment-as-usual with no neuropsychological intervention) in patients undergoing treatment for alcohol dependence syndrome. Sixty-eight patients were recruited from an alcohol-rehab clinic and randomly assigned to the mHealth (n=33) or control condition (n=35). The intervention on the experimental group consisted of a therapist-assisted cognitive stimulation therapy for 4 weeks on a 2-3 days/week basis.

**Results:**

Fourteen patients dropped out of the study. The results of the neuropsychological assessments with the remaining 54 patients showed an overall increase (*P*<.05) of general cognitive abilities, mental flexibility, psychomotor processing speed, and attentional ability in both experimental (n=26) and control groups (n=28). However, there was a more pronounced improvement (*P*=.01) specifically in frontal lobe functions from baseline (mean 13.89, SE 0.58) to follow-up (mean 15.50, SE 0.46) in the experimental group but not in the control group.

**Conclusions:**

The overall increase in general cognitive function for both experimental and control groups supports the beneficial role of existing alcohol treatment protocols aimed at minimizing withdrawal symptoms, but the differential improvements observed in frontal lobe functioning supports the use of mobile serious games for neuropsychological stimulation to overcome executive dysfunction in patients with alcohol dependence. This trial was negative on two neuropsychological/cognitive tests, and positive on one.

**Trial Registration:**

ClinicalTrials.gov NCT01942954; http://www.clinicaltrials.gov/ct2/show/NCT01942954 (Archived by WebCite at http://www.webcitation.org/6OYDqHLwB).

##  Introduction

Alcohol abuse is a global health problem and cause of excess mortality and morbidity, as well as a source of personal disruption for both abusers and their families. A representative survey-based study of the US population found that excessive consumption of alcohol increased risk of mortality over a 14-year period [1]. A study in Northern Germany concluded that alcohol dependency (as assessed by the Diagnostic and Statistical Manual of Mental Disorders, 4^th^ edition [DSM-IV]) increased the mortality rate almost two-fold for men and more than four-fold for women [2].

Apart from excess mortality, the negative effects of alcohol on the brain can also be severe. Alcohol dependence syndrome has been related to brain dysfunction specifically in the prefrontal cortex, which is associated with cognitive functioning [3]. In fact, the available evidence suggests that alcohol abuse produces a decrease in specific cognitive abilities, particularly those associated with executive functions [4]. Many studies have found a positive correlation between the integrity of white matter and cognitive performance in teenagers and a negative effect of prolonged alcohol dependence on the integrity of white matter in adults (eg, [5]). This suggests that alcohol use is related to the reduction of the integrity of grey matter, as the integrity of grey matter depends on functional white matter, in particular, the superior longitudinal fasciculus. Also, several studies with general population samples indicate that the prefrontal cortex is particularly vulnerable to the neurotoxic effects of alcohol [6]. A recent review of 62 papers assessing cognitive dysfunction in alcoholics concluded that (1) several cognitive functions become significantly compromised, and remain so, even during abstinence, with stable effects for 1 year of abstinence, (2) without intervention, these effects start reversing after 1 year of abstinence, (3) they appear to support the diffuse cerebral hypothesis, but (4) it is still unclear which cognitive functions are more susceptible to be compromised, as well as what the necessary timeframe is for cognitive recovery [7]. What is known is that there is a relation between the excessive consumption of alcohol and the compromising of cognitive functions in a variety of domains, namely, attention, working memory, processing speed, visual-spatial capacities, impulsivity, learning, memory, verbal fluency, decision making, and executive functions [8-14]. One study showed that, even when sober, binge drinking (BDs) university students performed significantly worse on planning tasks (they took significantly longer to plan an action) and on attention/working memory tasks (they got fewer correct answers on the Paced Auditory Serial Addition Task [PASAT]) than university students who did not binge drink (non-BDs) [15]. Another study found that BDs showed a lower capacity to retain and manipulate information in the verbal working memory (Digit Span Backward, Wechsler Memory Scale [WMS-III]), as well as a greater number of persevering responses (although only on the speed of processing test [SOPT], and not on the Wisconsin Card Sorting Test [WCST]) than non-BDs [16], indicating a lower capacity on executive tasks, which depend on the functional integrity of the prefrontal cortex. Another study also found that BD university students had worse results on perseverative and intrusion errors and more false positives (Complutense Verbal Learning Test [TAVEC]), as well as greater interference (Stroop tests) and worse performance in the Digit Span (WMS-III), Corsi Block Test, and the Series Recall Test [17].

In sum, patients with alcohol dependence are clearly an important target population for neuropsychological rehabilitation programs in general and cognitive rehabilitation programs in particular. However, the literature on the effectiveness of neuropsychological rehabilitation in alcohol addicts is still scarce. A few years ago, a review of cognitive rehabilitation programs with alcoholics noted that although patients’ responses to cognitive rehabilitation techniques were generally satisfactory, those techniques had not been used in traditional treatment programs [18]. Subsequent studies have shown that training and stimulating cognitive functions that have been compromised by the effects of drugs have some positive effects. For example, a study of 120 drug addicts in treatment found that patients benefiting from cognitive rehabilitation remained in treatment for significantly longer periods, and 38% of this group ended treatment successfully, compared to only 18% of other groups [19]. Another study with 60 drug addicts in in-residence treatment found that patients undergoing cognitive rehabilitation treatment became significantly more committed to the treatment, assimilated therapeutic content better, remained in abstinence for longer periods after ending the treatment, and had better scores on social and family behavioral normalization, as well as greater reduction of legal problems, than those who did not undergo treatment [20]. In addition, a study with 40 alcoholics found that a group subjected to cognitive rehabilitation significantly improved their level of information processing, visual-constructive abilities, and decision-making process compared to a group that did not undergo cognitive rehabilitation [21]. A recent review of all available studies on cognitive rehabilitation (CR) related to addiction concluded that there is a clear tendency for improvement of success rates in the treatment of patients that are targeted by specifically cognitive rehabilitation programs [22].

Despite the apparent added value of CR, the large majority of programs are still based on pencil-and-paper tasks with questionable ecological validity for functionality in daily life. Serious games (SG), that is, games designed for other purposes than gaming, seem to be a sound way to overcome this flaw by simulating real life activities or by simply challenging patients’ cognitive functions through an interactive and appealing interface. Several have already been designed and applied to stroke and traumatic brain injury rehabilitation (eg, [23,24]). SGs are usually platforms that encompass training environments where repetition and visual and auditory feedback can be systematically manipulated according to individual specificities. And because these are games, patients are usually motivated to execute the proposed exercises. Thus, SGs combine three important aspects that may contribute to the effectiveness of this approach: repetition, feedback, and motivation. Repetition refers to an intrinsic characteristic of games, which is the possibility of repeating over and over the same action in a pleasing way. Feedback refers to the fact that, while carrying out exercises, the patient’s senses are provided with feedback on the accomplishments achieved during each task. Finally, motivation is probably enhanced in SG because they are usually presented on a multimodal platform with different immersive cues, such as images and sounds, where patients may be more willing to engage and pursue an exercise.

The increasing development of both hardware and software has allowed the use of mobile devices in cognitive rehabilitation programs [23]. It is now possible to design, develop, and apply training and treatment programs over mobile health (mHealth) applications. Mobile device-based interventions are already used, for example, to enhance emotional awareness [25] or to treat emotional disorders such as depression [26] or anxiety [27], as self-help programs to reduce cocaine consumption [28], or to support patients with mild acquired cognitive impairments [29]. There are also some studies on alcohol abusers that reflect this shift of attention. For example, Hester et al developed a Web-based application so that alcoholics could overcome their drinking problems [30]. They found that patients using their program significantly decreased their average number of drinks per drinking day and alcohol-related problems, and also increased their average number of abstinence days. The application, however, did not produce better results than the traditional approach.

Another study compared the outcomes of two types of Web-based interventions on 170 problem drinkers. In one of the interventions, Check Your Drinking (CYD) [31], screened participants were invited to answer questions about the quantity and frequency of their drinking and with the severity of their drinking problems. In the other, the Alcohol Help Center (AHC) [32], participants had to undergo several exercises based on cognitive and behavioral principles, designed to increase motivation levels and prevent relapse. A significant additional reduction in drinking for participants in the AHC group was found when compared with the CYD group [33].

However, for addiction-related cognitive impairments, research assessing the effect of cognitive stimulation using mHealth SG programs for the improvement of cognitive functioning is still in its infancy. The objective of the current study was to assess the outcome of cognitive stimulation exercises over a Web platform developed to tackle cognitive impairments of individuals with Alcohol Dependence Syndrome (ADS) [34]. The potential impact of using such an approach relies on the possibility of democratizing the access and the usage of cognitive stimulation programs throughout the health care system (ie, both through caregivers and directly to patients), thus also reducing the societal burden associated with the costs of cognitive impairments and treatments.

##  Methods

### Trial Design

The study design consisted of a two-arm randomized controlled trial (RCT) developed to assess the neuropsychological effects of mHealth applications in alcoholics. (The trial was registered retrospectively because our funding agency, the Portuguese National Science Foundation [FCT] does not require registration for psychological tests nor does our home country have a registration system.) Our sample size was estimated based on a priori power analysis according to commonly accepted standards in the field (see Statistical Procedures) and agreed to in advance with the clinical institution where the trial took place.

The method of patient assignment was based on simple randomization with a random number generator. The patients were randomly assigned to experimental and control groups in a specialized institution for treatment of alcohol dependence (see Multimedia Appendix 1 for the CONSORT eHealth Checklist). The trial was approved by the Ethics Committee of the research center where the authors were affiliated at the time (Centre for the Study of Cognitive and Learning Psychology [CEPCA]) and adhered to the principles of the World Medical Association’s Declaration of Helsinki.

### Participants

Sixty-eight patients diagnosed with ADS according to DSM-IV criteria were recruited from a specialized institution for treatment of alcohol dependence, the Novo Rumo Clinic, São João de Deus Institute in the Lisbon region, Portugal, and were asked to participate in a study on the effects of their treatment on cognitive abilities. In the experimental condition, they were told that their treatment would include cognitive exercises. Due to a dropout rate of 20.6% (14/68 patients), the final sample consisted of 54 patients.

### Inclusion Criteria

Only patients that scored higher than the cut-off values for their age (see Outcomes) on the Mini Mental State Examination (MMSE) [35] and with no clinical scores on the Symptoms Checklist Revised (SCL-90-R) [36] were included in the study.

Patients continued during the entire study their regular medication regimen consisting of anxiolytics, mostly Diazepam and Tiapride, which help minimize withdrawal symptoms, and vitamins. Each patient’s assistant psychiatrist guaranteed the stability of the medication regimens throughout the program.

### Exclusion Criteria

Patients with dependency on substances other than alcohol or with a history of previous neurological disorders were excluded from the study. Patients were also screened for minimal computer literacy, but no patient was excluded due to this criterion (ie, all patients demonstrated the minimum literacy required).

### Study Procedure

After initial recruitment and screening, participants were randomly assigned to either the experimental group with mHealth SG-based cognitive stimulation plus treatment-as-usual (n*=*33) or to the control group (n*=*35), which received solely treatment-as-usual. The treatment-as-usual consisted of an alcohol-abstinence program adapted from the Minnesota Model (see [37] for a detailed description of this program) and lasted for an average period of 1 month.

**Figure 1:**
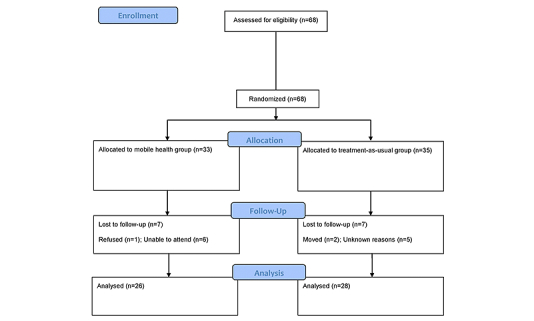
Flow chart describing the flow of the participants in the treatment group throughout the protocol.

Figure 1 illustrates the flow of participants in the experimental group throughout the protocol. Participants failing to complete training sessions within the assigned time-frame were considered to have dropped out and their data were not analyzed.

The trial took place over a period of 6 months, and no changes to the stimulus program were made during this period. Both the treatment and the assessments took place on location at the clinic where participants were recruited.

Each participant underwent two complete neuropsychological assessments, once after they had undergone screening and given their written informed consent to treatment and assessment, and again after at least 30 days. In the case of the treatment group, this was done after completing the intervention.

The intervention consisted of ten 60-minute sessions of cognitive stimulation with mobile technology using SG (2-3 sessions per week over the usual 4-6 week period of treatment). No institutional affiliations were presented in the eHealth media. The executive training exercises performed by participants in the experimental group were selected in order to develop cognitive abilities related to executive functioning. Each session started with a brief training period, when participants were able to (re)acquire interaction skills with the touchscreen devices. Participants accessed the exercises freely over the Internet, and their responses were registered using the input from the device’s touchscreen.

Therapists from the research and intervention team were involved in both the recruitment and the cognitive stimulation. These therapists were introduced to patients by in-house therapists and asked patients to participate in the study, explaining its benefits, duration, and demands on patients’ time and commitment. During the assessments, other therapists provided, explained, and collected the assessment forms. In the cognitive simulation sessions, the first group of therapists provided the mobile devices, launched the exercises, and explained how they worked to participants.

**Figure 2:**
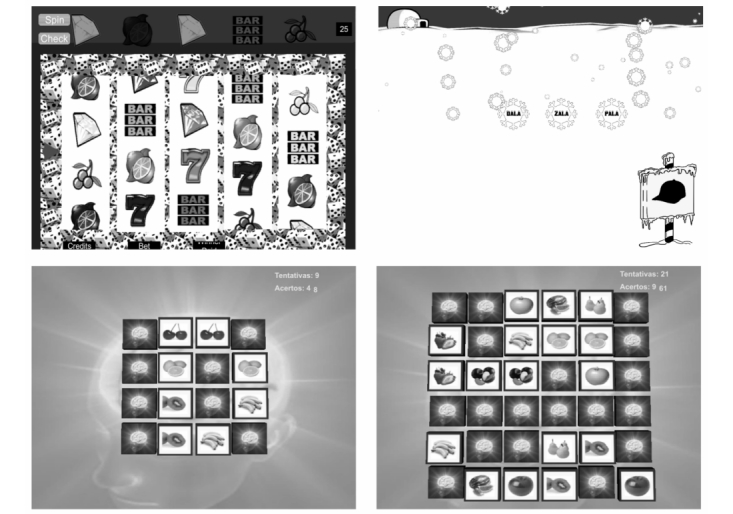
Slot machine (top-left) for attention; visual memory task (bottom-left and bottom-right with increased difficulty) for working memory; and word-object correspondence (top-right) for logical reasoning.

The mobile cognitive stimulation program consisted of several mobile applications developed to run on Android OS, which were adapted from the traditional paper-and-pencil exercise conceived for cognitive stimulation on patients with cognitive impairments, independently of the cause, but selected for their relevance to address the most common cognitive impairments in addicts. Cognitive stimulation in each session comprised attention, working memory, and logical reasoning exercises (see Textbox 1 for a more detailed description; these exercises (Figure 2) are available online [34]). The level of difficulty of each task increased progressively throughout the cognitive stimulation rationale. In the last session, the same neuropsychological tests used in the first assessment were again applied.

The hardware used to perform the exercises consisted of Samsung Galaxy 10.1ʺ tablets. The applications were developed using Unity 2.5, and their alpha and beta versions had been previously tested by a group of students.

**Cognitive stimulation program—sessions and mHealth applications. a: Perception; b: Processing speed; c: Reasoning; d: Attention; e: Memory; f: Decision making; g: Planning; h: Spatial vision.**

Session 1. Slot^d^/Memory^e^/Parking Zone^g^/Under pressure^h^/Snowflakes^b^

Session 2. Slot^d^/Memory^e^/Under pressure^h^/Snowflakes^b^/Right order^c^

Session 3. Slot^d^/Memory^e^/Hanoi Tower^f^/Snowflakes^b^/Right order^c^

Session 4. Slot^d^/Memory^e/^Odd-even^c^/Parking Zone^g^/Snowflakes^b^

Session 5. Basket^c^/Odd-even^c^/Hand tricks^h^/Brick^a^/Memory^e^

Session 6. Hanoi Tower^f^/Parking Zone^g^/Under pressure^h^/Memory^e^/Snowflakes^b^

Session 7. Parking Zone^g^/Under pressure^h^/Selective transfer^d^/Memory^e^/Snowflakes^b^

Session 8. Selective transfer^d^/Brick^a^/Hand tricks^h^/Memory^e^

Session 9. Parking Zone^g^/Brick^a^/Hand tricks^h^/Memory^e^

Session 10. Slot^d^/Memory^e^/Parking Zone^g^/Under pressure^h^/Snowflakes^b^

### Outcomes

All neuropsychological assessments were carried out with the pencil-and-paper forms of well-established cognitive tests. Global cognitive abilities were assessed with the MMSE [35], a brief screening test that has been validated for the Portuguese population [38]. The MMSE assesses diverse aspects of cognitive mental function, allowing an overall assessment of cognitive performance based on 30 items grouped into 6 categories: Orientation, Retention, Attention and Calculation, Language, and Visual-spatial abilities. The maximum score is 30 points and the current cut-off values for the Portuguese population were estimated according to age and education, specifically for people aged over 40 years: (1) 0-2 years of schooling—22, (2) 3-6 years of schooling—24, and (3) more than 7 years of schooling—27. MMSE was dictated on the basis of education.

Frontal brain functioning was evaluated with the Frontal Assessment Battery (FAB) [39]. The FAB assesses conceptualization, mental flexibility, motor programming, sensitivity to interference, inhibitory control, and environmental autonomy. The total score of the FAB (maximum 18 points) is estimated through the sum of each of the subtest scores. The FAB assesses 6 domains of frontal functioning through the ability to generate similarities (conceptualization), verbal fluency (mental flexibility), Luria’s motor series (motor programming), conflicting instructions (sensitivity to interference), go-no go paradigm (inhibitory control), and prehension behavior assessments (environmental autonomy).

Cognitive flexibility was measured with the WCST [40], which evaluates cognitive functions in several executive domains, namely the ability to develop and maintain appropriate strategies for problem solving and planning, and the ability to use environmental feedback to modify a cognitive response. The WCST assesses cognitive abilities such as abstraction, mental flexibility, and sustained attention. We focused on the overall index of performance through correct responses, categories, and perseverative errors. In the current study, we used the short version of the test (WCST-64).

Psychomotor processing speed and attentional abilities were estimated through the Color Trail Test (CTT) [41]. The CTT assesses focused and divided attention, sequencing, mental flexibility, visual search, and motor functions. The CTT consists of an A4 sheet containing circles with numbers printed in yellow and pink. Participants are asked to link the numbered circles in the right order, as fast as possible, and without lifting the pen. The CTT consists of two different forms (CTT1 and CTT2), which differ in difficulty. Two measures of performance were analyzed: errors and execution time.

### Statistical Analysis

The variables evaluated during the neuropsychological assessments were submitted to parametric statistical analyses. The dependent variables were based on the neuropsychological outcomes from the MMSE, FAB, WCST, and the CTT. Scores reported refer to before intervention (baseline) and after intervention (follow-up).

Sample and baseline characteristics were compared between the experimental and control groups with Student’s *t* test for independent samples for interval dependent variables and chi-square test for categorical dependent variables.

The statistical analyses of evaluation outcomes were based on generalized linear model procedures using type III Sum of Squares. A full factorial model (within-between interaction) was tested using repeated measures analysis of variance (ANOVA). The factorial design consisted of one within-subjects factor with 2 levels (baseline vs follow-up) and one between-subjects factor with 2 levels (experimental vs control group). Missing data imputation was completed using linear interpolation method. A significance level of .05 was adopted for all statistical procedures. Post-hoc effect sizes are eta-square in the ANOVA procedure, and within-between interactions were tested using simple effects.

Inferential statistics were carried out using IBM SPSS v.20. A priori power analysis was estimated with G*Power v.3.1 with Cohen’s *f* effect size for *F* tests [42].

### Sample Size

The expected effect size for calculating the required sample size was 0.35 (medium effect) based on a power of 0.80 for a significance level of .05. According to these criteria, a total sample size of 68 patients would be required for this trial. This was the initial size of the sample recruited for this study.

##  Results

### Sample Characteristics

Due to a dropout rate of 20.6% (14/68 patients), the final sample consisted of 54 patients diagnosed with alcohol dependence syndrome: 45 males and 9 females, mean age 45.37 years (SD 10.12) with an average of 10 years of formal education (SD 4.62). Of these, 26 patients had been assigned to the mobile health cognitive stimulation condition: 19 males and 7 females, mean age 45.50 years (SD 10.18) with an average of 10 years of education (SD 4.39). Twenty-eight patients were assigned to the control condition consisting of treatment-as-usual: 26 males and 2 females, mean age 45.25 years (SD 10.26) with an average of 10 years of formal education (SD 4.95). No statistically significant differences at the conventional *P*<.05 level were observed between groups for the demographic characteristics of gender (Table 1), age, and education (Table 2).

**Table 1:** Baseline demographics for categorical data.

Characteristics		mHealth, n (%)	Treatment-as-usual, n (%)	χ^2^	*P* value
**Gender**				3.798	.051
	Male	19 (35.2)	26 (48.1)		
	Female	7 (13.0)	2 (3.7)		

**Table 2:** Baseline demographics for interval data.

Characteristics	mHealth, mean (SD)	Treatment-as-usual, mean (SD)	*t*	*P* value
Age, yrs	45.50 (10.18)	45.25 (10.26)	0.090	.929
Education, yrs	10.18 (4.93)	10.25 (4.95)	–0.052	.958

### Baseline Characteristics

The statistical analyses for the neuropsychological data at baseline were focused on comparisons between groups for the overall score of the MMSE and the FAB, the correct responses, categories and perseverative errors in the WCST, and the mean errors and execution times in the CTT. The results revealed no statistically significant differences between the experimental and control group for neuropsychological outcomes at baseline (all *P*s>.41) (Table 3).

**Table 3:** Baseline characteristics for both mHealth and control.

Characteristics		mHealth, mean (SD)	Treatment-as-usual, mean (SD)	*t*	*P* value
**MMSE**					
	Total score	26.46 (2.97)	26.38 (2.67)	0.098	.922
**FAB**					
	Total score	13.89 (2.86)	14.14 (3.05)	–0.320	.750
**WCST**					
	Correct responses	52.85 (20.05)	52.48 (17.01)	0.068	.946
	Categories	54.86 (35.60)	50.00 (35.27)	0.485	.630
	Perseverative errors	24.78 (15.30)	25.57 (13.56)	–0.193	.848
**CTT1**					
	Errors	0.23 (0.58)	0.26 (0.08)	–0.146	.409
	Execution time (seconds)	86.65 (42.45)	114.02 (162.60)	–0.832	.885
**CTT2**					
	Errors	0.81 (1.09)	0.85 (1.29)	–0.134	.894
	Execution time (seconds)	173.42 (82.06)	169.71 (93.44)	0.154	.878

### Evaluation Outcomes

**Figure 3:**
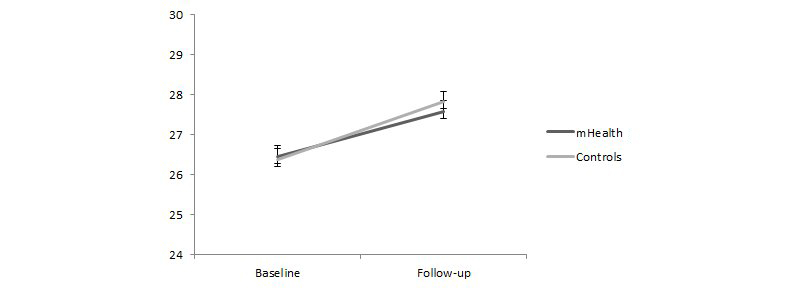
MMSE total scores for the experimental and control conditions at baseline and follow-up.

Global cognitive function was estimated at baseline and follow-up assessment with the MMSE. The ANOVA on the overall score of the MMSE revealed a main effect of assessment (*F*_1,52_=20.68, eta-square=0.41, *P*<.001), indicating an improvement of cognitive ability from baseline (mean 26.42, SE 0.38) to follow-up (mean 27.70, SE 0.26). No statistically significant main effect of group or interaction effect between factors (*F*s<1) was found (Figure 3).

**Figure 4:**
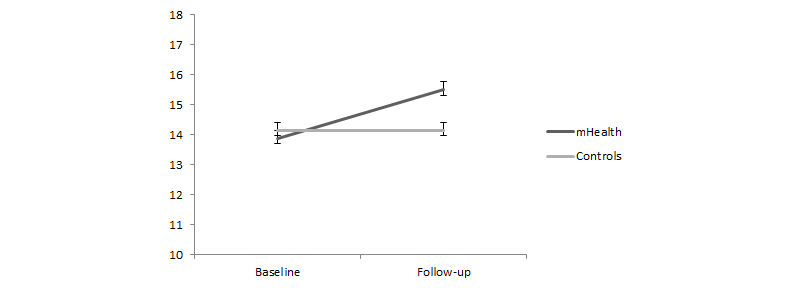
FAB total scores for the experimental and control conditions at baseline and follow-up.

Frontal lobe functions were also evaluated under the same factorial design. The data from the FAB showed a significant interaction effect between factors (*F*_1,52_=8.00, eta-square=0.16, *P*=.01). Simple effects analysis was performed to test differences between assessments on each group (experimental and control). These data indicated a significant increase in the FAB score from baseline (mean 13.89, SE 0.58) to follow-up (mean 15.50, SE 0.46) only in the experimental group, but not in the control group (mean 14.14, SE 0.56) and (mean 14.14, SE 0.45), respectively for baseline and follow-up (Figure 4).

**Figure 5:**
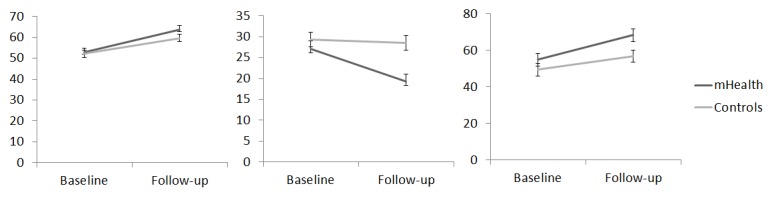
WCST correct responses (left) perseverative errors (middle) and completed categories (right) for the experimental and control conditions at baseline and follow-up.

Cognitive flexibility was measured on three different dimensions of the WCST: the correct responses, the number of categories, and the number of perseverative errors during the task. The ANOVA showed a main effect of assessment on the number of correct responses (*F*_1,52_=15.10, eta-square=0.29, *P*<.001) and on the number of completed categories (*F*_1,52_=4.94, eta-square=0.09, *P*=.03), indicating an improvement in performance of the WCST from baseline to follow-up assessment, but there was no interaction effect with the treatment factor (*F*<1). No significant effects (*F*<1) were reported for perseverative errors (Figure 5).

**Figure 6:**
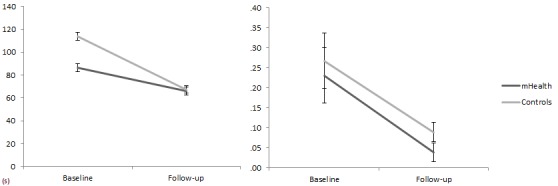
CTT1 execution time in seconds (left) and mean errors (right) for the experimental and control conditions at baseline and follow-up.

**Figure 7:**
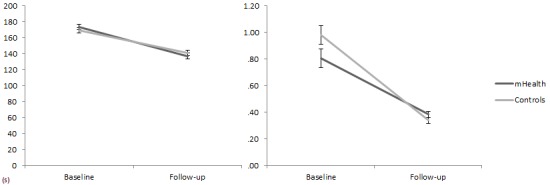
CTT2 execution time in seconds (left) and mean errors (right) for the experimental and control conditions at baseline and follow-up.

Speed of processing and attentional functioning were assessed respectively with execution time and error rate of the two forms of the CTT. We found a main effect of assessment for both error rate and execution time in the CTT1 and in the CTT2. A significant decrease in error rate (*F*_1,52_=5.20, eta-square=0.10, *P*=.03) and execution time (*F*_1,52_=4.26, eta-square=0.08, *P*=.04) was found in the CTT1 (Figure 6). The same pattern, but with a stronger effect, was found for the results of the CTT2, with a decrease in error rate (*F*_1,52_=13.23, eta-square=0.25; *P*<.001) as well as execution time (*F*_1,52_=14.41, eta-square=0.28, *P*<.001) (Figure 7). In both the CTT1 and the CTT2, there was no significant interaction (*F*<1) with the treatment factor.

## Discussion

### Principal Findings

The current experiment was designed to study the effectiveness of a cognitive stimulation program in alcohol abusers’ executive functioning with mobile SG applications. According to previous studies, alcohol dependence may impair information processing in the prefrontal areas, such as the dorsolateral prefrontal cortex and the anterior cingulate cortex, which are thought to be related to executive functioning in cognitive domains ranging from attention and working memory to higher-order functions of reasoning and decision making. In this study, our aim was to evaluate the effect of cognitive stimulation with mobile technology and serious games on the general cognitive ability of patients diagnosed with ADS.

To do this, we carried out an RCT on patients undergoing an alcohol rehabilitation treatment at a specialized institution for treatment of alcohol dependence. The overall MMSE scores at baseline suggest deficits in general cognitive ability of ADS patients, which concurs with previous studies highlighting the relationship between the alcohol abuse and cognitive impairments [4]. There were no differences at baseline between control and treatment groups, which indicates a successful random distribution of patients.

Our results indicate a general improvement of cognitive abilities in both patient groups, which reflects an effect of the withdrawal of the direct impact of alcohol on the brain. In support of the specific effectiveness of the cognitive stimulation, this effect was qualified with an interaction with the treatment factor in the FAB test, indicating a more pronounced increase of frontal cognitive abilities in patients subjected to mHealth SG approach, with improvements in frontal lobe function and executive functioning. However, there were no significant effects of the treatment on the MMSE and WCST, which measure more specific, not general, domains of frontal lobe functioning. These results are in agreement with those of other cognitive rehabilitation trials with addict populations, suggesting that the positive effects of neuropsychological rehabilitation in addicted populations are restricted to cognitive abilities related to frontal lobes [21].

The explanation for this is that both traditional exercises and SG applications focused mainly on cognitive abilities related to frontal lobe functioning, such as attention, working memory, decision making, and planning. The systematic and repeated stimulation of these functions may have had a more pronounced effect on those domains, but not on the others. Our results suggest that the neuropsychological benefits of our mHealth SG cognitive stimulation program in alcoholics may be limited to frontal lobe general functioning and that may arise from the response demands of the game tasks. That is, the processes required to meet the games’ tasks are most closely related to the processes that are captured by the FAB.

In addition, the overall feedback from the participants was positive. Qualitative comments were mainly related to the technological and innovative features of this approach and intrinsic aspects such as a positive motivation to pursue a goal in the tasks.

### Limitations

This study was not designed to test the relative effectiveness of mobile intervention with online stimulation and serious games against traditional pencil-and-paper cognitive stimulation exercises. It was simply to test if this mHealth solution could add effectiveness in cognitive rehabilitation to treatment-as-usual procedures. Further studies testing the relative effectiveness of SG vs traditional approaches are needed. Due to the characteristics of SG and of the clinical context, it would not have been possible to conduct a double blind study. However, the therapists conducting the assessment were not aware of which group (experimental vs control) the patients were enrolled in.

### Conclusions

The increase in general cognitive function for both experimental and controls supports the beneficial role of existing alcohol treatment protocols, which are based on medication to help minimize withdrawal symptoms along with behavioral therapy mainly to help patients manage their stress levels during abstinence, and indicates that the neuropsychological effects of alcohol abuse on brain structure are reversible through rehabilitation. The effect of the trial is encouraging, suggesting an improvement specifically in frontal cognitive general functioning in alcoholics following an mHealth approach with SG in line with previous studies using cognitive stimulation with addicts in rehab.

## Multimedia Appendix 1

CONSORT-EHEALTH checklist V1.6.2 [43].

## Abbreviations

ADS: Alcohol Dependence Syndrome
AHC: AlcoholHelpCenter.net
ANOVA: analysis of variance
BD: binge drinking
CR: cognitive rehabilitation
CTT: Color Trail Test
CYD: Check Your Drinking
DSM-IV: Diagnostic and Statistical Manual of Mental Disorders, 4th edition
FAB: Frontal Assessment Battery
mHealth: mobile health
MMSE: Mini Mental State Examination
PASAT: Paced Auditory Serial Addition Task
RCT: randomized controlled trial
SCL-90-R: Symptoms Checklist Revised
SG: serious games
SOPT: Speed of Processing Test
SPSS: Statistical Package for Social Sciences
TAVEC: Verbal Learning Test of the Complutense University
WCST: Wisconsin Card Sorting Test
WMS: Wechsler Memory Scale
