# Supplementary material for: Executive Functioning in Alcoholics Following an mHealth Cognitive Stimulation Program: Randomized Controlled Trial
Source: J Med Internet Res. 2014 Apr 17;16(4):e102. doi: 10.2196/jmir.2923 (PMC4019779; doi:10.2196/jmir.2923)
Supplement: Supplementary file 1 [file jmir_v16i4e102_app1.pdf]

CONSORT-EHEALTH Checklist V1.6.2 Report

(based on CONSORT-EHEALTH V1.6), available at [http://tinyurl.com/consort-ehealth-v1-6].

2923

Date completed

8/30/2013 21:53:15

by

Pedro Gamito

Executive Functioning in Alcoholics Following a Mobile Health Cognitive Stimulation Approach: a Randomized Controlled Trial

TITLE

1a-i) Identify the mode of delivery in the title

Yes: "Following a Mobile Health Cognitive Stimulation Approach"

1a-ii) Non-web-based components or important co-interventions in title

This is not included in the title because the study did not use other forms of intervention

1a-iii) Primary condition or target group in the title

Yes: "in Alcoholics"

ABSTRACT

1b-i) Key features/functionalities/components of the intervention and comparator in the METHODS section of the ABSTRACT

Yes: "the cognitive outcomes of neuropsychological intervention with mobile serious games vs. control (no neuropsychological intervention)"

1b-ii) Level of human involvement in the METHODS section of the ABSTRACT

Yes: "intervention (...) consisted of a therapist-assisted cognitive stimulation therapy"

1b-iii) Open vs. closed, web-based (self-assessment) vs. face-to-face assessments in the METHODS section of the ABSTRACT

Yes: "patients (...) were recruited from an alcohol-rehab clinic"

1b-iv) RESULTS section in abstract must contain use data

Yes: Abstract refers to dropout rate: "14 patients dropped out of the study."

1b-v) CONCLUSIONS/DISCUSSION in abstract for negative trials

Yes: "The trial was negative on two neuropsychological/cognitive tests, and positive on one."

INTRODUCTION

2a-i) Problem and the type of system/solution

Yes. Two problems are described: the need for cognitive rehabilitation of patients with alcohol dependence, who suffer from reduced cognitive performance, and the need for appealing, interactive, and logistically efficient means (eg mobile e-health systems).

2a-ii) Scientific background, rationale: What is known about the (type of) system

Yes: "Serious games (SG) i.e. games that were designed for other purposes than gaming, seem to be a sound way to overcome this flaw by surrogating real life activities or by simply challenging patients' cognitive functions through an interactive and appealing interface."

METHODS

3a) CONSORT: Description of trial design (such as parallel, factorial) including allocation ratio

Yes: "The aim of the study reported here was to test the effects of a cognitive stimulation program with mobile SG applications on the recovery of executive functions in alcohol abusers undergoing rehabilitation."

3b) CONSORT: Important changes to methods after trial commencement (such as eligibility criteria), with reasons

Yes.

3b-i) Bug fixes, Downtimes, Content Changes

Yes. We indicate in 'Study procedure' that "The trial took place between October 2012 and March 2013, and no changes to the stimulus program were made during this period."

4a) CONSORT: Eligibility criteria for participants

Yes.

4a-i) Computer / Internet literacy

Yes. We indicate in Exclusion Criteria that "Patients were also screened for minimal computer literacy; no patients were excluded due to lack of this criterion."

4a-ii) Open vs. closed, web-based vs. face-to-face assessments:

Yes. In 'Participants' we mention that "68 patients were recruited from a specialized institution for treatment of alcohol dependence" and in 'Study procedure' we explain that "Therapists from the research and intervention team were involved in all stages of the study involving the participants, interacting face-to-face with them: recruitment, assessment, and cognitive stimulation. These therapists were introduced to patients by in-house therapists, and asked patients to participate in the study, explaining its benefits, duration, and demands on patients' time and commitment. In the assessments, therapists provided, explained, and collected the assessment forms. In the cognitive simulation sessions, therapists provided the mobile devices, opened the exercises, and explained how they worked to participants."

4a-iii) Information giving during recruitment

Yes. In 'Participants' we indicate that "(participants) were recruited from a specialized institution for treatment of alcohol dependence, the Novo Rumo Clinic – São João de Deus Institute in the Lisbon region, Portugal, and asked to participate in a study on the effect of their treatment on cognitive abilities. In the treatment condition, they were told that their treatment would include cognitive exercises." In 'Study procedure' we also indicate that they started their first assessment after signing the written consent form. This is provided in Appendix 2.

4b) CONSORT: Settings and locations where the data were collected

Yes.

4b-i) Report if outcomes were (self-)assessed through online questionnaires

Yes. In 'Outcomes' we report that "All neuropsychological assessments were carried out with pencil-and-paper forms of well-established cognitive tests." and, in the 'Study procedure' we indicate that "Both the treatment and the assessments took place on location at the clinic where participants were recruited."

4b-ii) Report how institutional affiliations are displayed

Yes. In the 'Study procedure' we note that "No institutional affiliations were presented in the e-health media."

5) CONSORT: Describe the interventions for each group with sufficient details to allow replication, including how and when they were actually administered

5-i) Mention names, credential, affiliations of the developers, sponsors, and owners

Yes. As reported in 'Conflicts of interest': "The authors owned and have developed the majority of the applications. These are freely available and no commercial profit is intended from them."

5-ii) Describe the history/development process
Yes. In the Study Procedure we mention "The applications were developed using Unity 2.5 (Unity Technologies TM), and their alpha and beta versions had been previously tested by a group of students."
5-iii) Revisions and updating
Yes.
5-iv) Quality assurance methods

5-v) Ensure replicability by publishing the source code, and/or providing screenshots/screen-capture video, and/or providing flowcharts of the algorithms used
Yes. It is available online at URL:http://labpsicom.ulusofona.pt. Accessed: 2013-08-29. Archived by WebCite® at http://www.webcitation.org/6JEYtW6JH.
5-vi) Digital preservation
Yes: We included a reference for the online platform: PedroGamito. Professor. . 2013-08-29. URL:http://labpsicom.ulusofona.pt. Accessed: 2013-08-29. Archived by WebCite® at http://www.webcitation.org/6JEYtW6JH.
5-vii) Access
Yes: In 'Study Procedure' we explain that "Both the treatment and the assessments took place on location at the clinic where participants were recruited." (paragraph 1) and that "In the assessments, therapists provided, explained, and collected the assessment forms. In the cognitive simulation sessions, therapists provided the mobile devices, opened the exercises, and explained how they worked to participants."
5-viii) Mode of delivery, features/functionalities/components of the intervention and comparator, and the theoretical framework
Yes: This is reported in the Study Procedure: "The mobile cognitive stimulation program consisted of several mobile applications running on Android OS that were developed according to traditional paper-and-pencil rationales and originally conceived for cognitive stimulation on patients that had acquired cognitive impairments independently of the cause. Cognitive stimulation in each session comprised attention, working memory and logical reasoning exercises (see Textbox 1 for a more detailed description). The level of difficulty of each task increased progressively throughout the cognitive stimulation rationale. In the last session, the same neuropsychological tests used in the first assessment were again applied."
5-ix) Describe use parameters
As reported in the Study Procedure, "The intervention consisted of 10 60-minutes sessions of cognitive stimulation with mobile technology (two to three sessions per week over the usual 4-6 week period of treatment)."
5-x) Clarify the level of human involvement
This is explained in the Study procedure, 4th paragraph: "Therapists from the research and intervention team were involved in all stages of the study involving the participants, interacting face-to-face with them: recruitment, assessment, and cognitive stimulation. These therapists were introduced to patients by in-house therapists, and asked patients to participate in the study, explaining its benefits, duration, and demands on patients' time and commitment. In the assessments, therapists provided, explained, and collected the assessment forms. In the cognitive simulation sessions, therapists provided the mobile devices, fired up the exercises, and explained how they worked to participants."
5-xi) Report any prompts/reminders used
Prompts were not required as all training sessions were with therapist present (see 5x)
5-xii) Describe any co-interventions (incl. training/support)
Yes: The control condition consisted of treatment-as-usual in an alcohol-abstinence program according to the Minnesota Model within the context of a therapeutic community [see Study Procedure).
6a) CONSORT: Completely defined pre-specified primary and secondary outcome measures, including how and when they were assessed
Yes: Outcomes were all measured offline, as indicated in the Study Procedure
6a-i) Online questionnaires: describe if they were validated for online use and apply CHERRIES items to describe how the questionnaires were designed/deployed

6a-ii) Describe whether and how "use" (including intensity of use/dosage) was defined/measured/monitored

6a-iii) Describe whether, how, and when qualitative feedback from participants was obtained

6b) CONSORT: Any changes to trial outcomes after the trial commenced, with reasons
Yes: There were no changes to trial outcomes after trials commenced
7a) CONSORT: How sample size was determined
7a-i) Describe whether and how expected attrition was taken into account when calculating the sample size
Expected sample attrition was not taken into account.
7b) CONSORT: When applicable, explanation of any interim analyses and stopping guidelines
Not applicable
8a) CONSORT: Method used to generate the random allocation sequence
Yes: The method of patient assignment was based on simple randomization with random number generator (see Trial Design in the Method section)
8b) CONSORT: Type of randomisation; details of any restriction (such as blocking and block size)
Yes: The method of patient assignment was based on simple randomization with random number generator (see Trial Design in the Method section)
9) CONSORT: Mechanism used to implement the random allocation sequence (such as sequentially numbered containers), describing any steps taken to conceal the sequence until interventions were assigned
Not applicable
10) CONSORT: Who generated the random allocation sequence, who enrolled participants, and who assigned participants to interventions
Investigators
11a) CONSORT: Blinding - If done, who was blinded after assignment to interventions (for example, participants, care providers, those assessing outcomes) and how
11a-i) Specify who was blinded, and who wasn't
Yes: This study was a single-blind study, i.e. the outcomes assessor was blind to the experimental group of participants. Patients could not be blinded, because controls were not submitted to a neutral mobile health condition (see Limitations in the Discussion)
11a-ii) Discuss e.g., whether participants knew which intervention was the "intervention of interest" and which one was the "comparator"
Yes: The participants could not be blinded to intervention.
11b) CONSORT: If relevant, description of the similarity of interventions
Not applicable
12a) CONSORT: Statistical methods used to compare groups for primary and secondary outcomes

Yes.
12a-i) Imputation techniques to deal with attrition / missing values
Yes: Participants failing to complete training sessions within the assigned time-frame were considered to drop out and their data was not analysed (see Study Procedure)
12b) CONSORT: Methods for additional analyses, such as subgroup analyses and adjusted analyses
Not applicable

RESULTS

13a) CONSORT: For each group, the numbers of participants who were randomly assigned, received intended treatment, and were analysed for the primary outcome
Yes. Patients were treated in a specialized institution for alcohol dependence treatment.
13b) CONSORT: For each group, losses and exclusions after randomisation, together with reasons
Yes. Described in then flow diagram.
13b-i) Attrition diagram
Yes. In the flow diagram.
14a) CONSORT: Dates defining the periods of recruitment and follow-up
Yes. Described in the method section "The trial took place between October 2012 and March 2013".
14a-i) Indicate if critical “secular events” fell into the study period
Not applicable
14b) CONSORT: Why the trial ended or was stopped (early)
Not applicable
15) CONSORT: A table showing baseline demographic and clinical characteristics for each group
Yes.
15-i) Report demographics associated with digital divide issues
Yes. Age, gender and education are presented in demographics section of the Results.
16a) CONSORT: For each group, number of participants (denominator) included in each analysis and whether the analysis was by original assigned groups
16-i) Report multiple “denominators” and provide definitions
Only data from participants who underwent full program and completed both baseline and outcome assessment was analysed. Group n’s refer to these participants (see Study Procedure)
16-ii) Primary analysis should be intent-to-treat

17a) CONSORT: For each primary and secondary outcome, results for each group, and the estimated effect size and its precision (such as 95% confidence interval)
Effect sizes and p levels were reported for all analyses. Lengths of training sessions per participant were not recorded, but ranged from c. 50 to 70min (c. 60mins)
17a-i) Presentation of process outcomes such as metrics of use and intensity of use

17b) CONSORT: For binary outcomes, presentation of both absolute and relative effect sizes is recommended
Not applicable
18) CONSORT: Results of any other analyses performed, including subgroup analyses and adjusted analyses, distinguishing pre-specified from exploratory
Not applicable
18-i) Subgroup analysis of comparing only users
Not applicable (no users-only analyses were carried out)
19) CONSORT: All important harms or unintended effects in each group
Not detected.
19-i) Include privacy breaches, technical problems
No privacy breaches were detected.
19-ii) Include qualitative feedback from participants or observations from staff/researchers
Yes: We described in the Discussion "In addition, the overall feedback from the participants was positive. Qualitative comments were mainly related to the technological and innovative features of this approach and intrinsic aspects such as a positive motivation to pursue a goal in the tasks."

DISCUSSION

20) CONSORT: Trial limitations, addressing sources of potential bias, imprecision, multiplicity of analyses
20-i) Typical limitations in ehealth trials
This is done in the Limitations section
21) CONSORT: Generalisability (external validity, applicability) of the trial findings
21-i) Generalizability to other populations
See 5viii
21-ii) Discuss if there were elements in the RCT that would be different in a routine application setting
This rct was carried out already within the context of a normal therapeutic community.
22) CONSORT: Interpretation consistent with results, balancing benefits and harms, and considering other relevant evidence
22-i) Restate study questions and summarize the answers suggested by the data, starting with primary outcomes and process outcomes (use)
This is the substance of both of the paragraphs of the discussion
22-ii) Highlight unanswered new questions, suggest future research
This is done in the Limitations section: “Further studies testing this relative effectiveness are warranted.”

Other information

23) CONSORT: Registration number and name of trial registry
The registry was done in clinicaltrial.gov database.
24) CONSORT: Where the full trial protocol can be accessed, if available
Yes. In the URL:http://labpsicom.ulusofona.pt. Accessed: 2013-08-29. (Archived by WebCite® at http://www.webcitation.org/6JEYtW6JH)
25) CONSORT: Sources of funding and other support (such as supply of drugs), role of funders

Yes. In Acknowledgements we state that "Hardware and software acquisition was funded by Centre for the Studies on Cognitive and Learning Psychology via the Foundation for Science and Technology – FCT of Portugal (PEst-OE/PSI/UI0700/2011)."

X26-i) Comment on ethics committee approval

Yes.

x26-ii) Outline informed consent procedures

Yes.

X26-iii) Safety and security procedures

Yes. Through training as described in the Method section.

X27-i) State the relation of the study team towards the system being evaluated

Yes.
